# Correction: Huang, H.-M., et al. Effects of *Sapindus mukorossi* Seed Oil on Skin Wound Healing: In Vivo and In Vitro Testing. *Int. J. Mol. Sci.* 2019, *20*, 2579

**DOI:** 10.3390/ijms20174178

**Published:** 2019-08-26

**Authors:** Chang-Chih Chen, Chia-Jen Nien, Lih-Geeng Chen, Kuen-Yu Huang, Wei-Jen Chang, Haw-Ming Huang

**Affiliations:** 1 Emergency Department, Mackay Momorial Hospital, Taipei 110, Taiwan; 2 Medical School, Mackay Medical College, New Taipei City 252, Taiwan; 3 Graduate Institute of Biomedical Optomechatronics, College of Biomedical Engineering, Taipei Medical University, Taipei 110, Taiwan; 4 Department of Microbiology, Immunology and Biopharmaceuticals, College of Life Sciences, National Chiayi University, Chiayi 600, Taiwan; 5 School of Dentistry, College of Oral Medicine, Taipei Medical University, Taipei 110, Taiwan

The authors are sorry to report that some of the HPLC data reported in their recently published paper [1] were incorrect. The data for β-sitosterol and δ-tocopherol was mismatched. In addition, the data of β-sitosterol were obtained from a 1% oil sample. Consequently, the authors wish to make the following corrections to the paper: On page 4, Section 2.2 HPLC Analysis, line 4, and page 12, Conclusion, line 5: The total amount of β-sitosterol and δ-tocopherol in the *S. mukorossi* seed oil was 73.9 ± 23.6 μg/mL (in 1% oil) and 232.64 ± 4.5 μg/mL, respectively.

There was one calculation error when the unit was changed from “μg/mL” to “%”: On page 8, paragraph 4, line 7, the sentence “*S. mukorossi* seed oil are 7.3% and 0.235%, respectively. These values are much higher than the analogous quantities in shea butter (0.8% for sterols and 0.08% for tocopherols)” should be “*S. mukorossi* seed oil are 0.73% and 0.023%, respectively. These values are much higher than the analogous quantities in shea butter (0.5% for total sterols and 0.08% for total tocopherols)”.

These changes have no material impact on the conclusions of our paper. We apologize to our readers.
